# MicroRNA Regulation of the Environmental Impact on Adolescent Neurobehavioral Development: A Systematic Review

**DOI:** 10.3389/fncel.2022.956609

**Published:** 2022-07-22

**Authors:** Ana Vázquez-Ágredos, Fernando Gámiz, Milagros Gallo

**Affiliations:** Department of Psychobiology, Institute of Neurosciences (CIBM), University of Granada, Granada, Spain

**Keywords:** adolescence, alcohol, behavior, brain, drug, epigenetic, microRNA, stress

## Abstract

Adolescence is a late developmental period marked by pronounced reorganization of brain networks in which epigenetic mechanisms play a fundamental role. This brain remodeling is associated with a peculiar behavior characterized by novelty seeking and risky activities such as alcohol and drug abuse, which is associated with increased susceptibility to stress. Hence, adolescence is a vulnerable postnatal period since short- and long-term deleterious effects of alcohol drinking and drug abuse are a serious worldwide public health concern. Among several other consequences, it has been proposed that exposure to stress, alcohol, or other drugs disrupts epigenetic mechanisms mediated by small non-coding microRNAs (miRNAs). During adolescence, this modifies the expression of a variety of genes involved in neurodevelopmental processes such as proliferation, differentiation, synaptogenesis, neural plasticity, and apoptosis. Hence, the effect of miRNAs dysregulation during adolescence might contribute to a long-term impact on brain function. This systematic review focuses on the miRNA expression patterns in the adolescent rodent brain with special interest in the impact of stress and drugs such as amphetamine, cocaine, nicotine, cannabis, and ketamine. The results point to a relevant and complex role of miRNAs in the regulation of the molecular processes involved in adolescent brain development as part of a dynamic epigenetic network sensitive to environmental events with distinctive changes across adolescence. Several miRNAs have been assessed evidencing changing expression profiles during the adolescent transition which are altered by exposure to stress and drug abuse. Since this is an emerging rapidly growing field, updating the present knowledge will contribute to improving our understanding of the epigenetic regulation mechanisms involved in the neurodevelopmental changes responsible for adolescent behavior. It can be expected that increased knowledge of the molecular mechanisms mediating the effect of environmental threats during the adolescent critical developmental period will improve understanding of psychiatric and addictive disorders emerging at this stage.

## Introduction

Adolescence is a late postnatal developmental period marked by the transition from infantile dependence on parental protection to adult independence. The peculiar adolescent behavior contributes to the acquisition of skills and resources required for independent survival. Accordingly, it seems to be highly conserved across mammals, with rodents being one of the most studied models (Spear, 2000; Varlinskaya and Spear, 2006). In comparison with an adult, adolescent behavior is not only characterized by a combination of novelty seeking but also by increased stress responsivity (Spear, 2013). Novelty seeking helps to explore novel peers, environments, and foods as required during this period. However, this is often dangerous as adolescents could be faced with risky stressful situations. Enhanced arousal and stress reactivity might help to solve these challenges by either fighting or flying more efficiently than adults. Therefore, it can be proposed that increased stress reactivity has short-term adaptive value to successfully cope with potential risks associated with novel experiences. On the contrary, enhanced adolescent stress reactivity is also associated with adverse long-term consequences. Accordingly, a major concern during adolescence is the use and abuse of alcohol and other drugs which is prompted both by sensation seeking and increased susceptibility to stress. In fact, a relationship between early life stress, defined as childhood maltreatment, and risk for alcohol and drug use disorders starting in adolescence has been proposed (Kirsch and Lippard, 2022). Both human and animal studies support an association between high levels of early life stress and increased risk of abuse across a broad range of substances, including alcohol, amphetamine, nicotine, cannabis, and cocaine, among others. This is a major health concern due to the potential adverse consequences on brain development, thus rendering the adolescent more vulnerable to addictive and psychopathological disorders (Spear, 2011). Moreover, stress and abuse of substances might exacerbate symptoms associated with psychiatric disorders such as schizophrenia (Thomas and Zakharenko, 2021) and depression (Morgunova and Flores, 2021). This is consistent with the high incidence of mental disorders is high during adolescence. Enhanced psychiatric vulnerability can be related to the great remodeling of the brain circuits that takes place during this developmental period (Spear, 2011).

The relationship established during adolescence between stress and heavy substance use, such as alcohol binge drinking, might be explained by neurodevelopmental changes. Adolescence is a postnatal developmental period in which synaptic pruning, epigenetic changes (Mychasiuk and Metz, 2016), and other maturational processes continue sculpting the brain formation (Spear, 2013). Due to different courses of the cortical and subcortical brain area maturation, a pronounced reorganization of reward and emotional brain networks takes place. Thus, changes in the mesocorticolimbic system might lead to reduced sensitivity to the rewarding effects of alcohol and other drugs, thus leading to heavier consumption. Also, modifications of the hippocampal and the amygdala (AM) circuits regulating stress throughout the hypothalamic–pituitary–adrenal (HPA) axis might enhance sensitivity to stress. This might predispose adolescents to increase alcohol and drug abuse to cope with it.

Cellular and molecular mechanisms involved in the association between stress and substance abuse during adolescence are not known. It can be proposed that it would rely on shared molecular mechanisms modulated by epigenetic processes which play a crucial role during brain maturation. According to current definitions, epigenetics refers to the regulation of gene expression by environmental factors independently of DNA sequences. A variety of epigenetic processes, including DNA methylation, histone modifications, and non-coding RNAs (ncRNAs), are modified by stress and drugs of abuse during adolescence (Mychasiuk and Metz, 2016). Among ncRNAs, interest in microRNA (miRNA) is rapidly increasing in the last years (Leighton and Bredy, 2018; Arzua et al., 2021). MicroRNAs are small ncRNAs that regulate messenger RNA (mRNA) translation by binding to the complementary mRNA sequence of target genes. This usually results in reduced gene expression by transcript degradation and/or prevention of mRNA translation, although increased mRNA translation has also been described (Rao and Pack, 2017; Morgunova and Flores, 2021). A single miRNA can bind to multiple mRNA targets resulting in the repression of hundreds of genes, including those coding enzymes involved in other epigenetic processes, as well as a single mRNA can be the target of multiple miRNAs. This can produce widespread and complex effects on genetic expression affecting brain development and function.

Even though miRNA levels vary with age (Thomas and Zakharenko, 2021), it is not clear, however, the role of miRNA expression in brain maturation during adolescence (Rao and Pack, 2017). The relevance of miRNAs mediating the environmental influence impact on neurodevelopment is suggested by clinical and preclinical studies. The available reviews focus on alcohol (Asimes et al., 2019; Zhu et al., 2022), nicotine and cocaine (Hayase, 2017), and stress related to psychiatric disorders risk (Morgunova and Flores, 2021). There is a recent review relating ncRNAs with the impact of prenatal stress, viral infections, and drugs, such as alcohol, nicotine, and anesthetics, during brain development (Leighton and Bredy, 2018; Arzua et al., 2021). The authors review both clinical and preclinical studies with special emphasis on early exposure. However, systematic reviews focused on the effects of stress and a variety of substances of abuse on the adolescent developmental period are lacking. Given the fact that there is a variety of ncRNAs with different functions, being miRNAs the best known and rodents the most abundant species studied, an update restricted to miRNAs and rodent studies could help to establish precise comparisons between the mechanisms involved. Hence, we have performed a systematic review to update knowledge obtained in rodent models on miRNA involvement in adolescent brain and behavior development and the influence of both stress and drugs of abuse such as amphetamine, cocaine, nicotine, cannabis, ketamine, and alcohol.

## Methods

### Design

This systematic review was conducted according to Preferred Reporting Items for Systematic Reviews and Meta-Analyses (PRISMA) statement guidelines (Page et al., 2021). We have included peer-reviewed research articles that explore the effect of stress, alcohol, and other drugs of abuse in adolescence on miRNA expression levels in the brain. Our analysis has focused on rodent models (rats and mice) that received either alcohol, stress, or other drug treatments during adolescence. Therefore, a systematic literature search was conducted in March 2022 using the databases Scopus and Web of Science (WOS).

### Inclusion and Exclusion Criteria

The inclusion criteria to select the studies included in this systematic review were as follows:

1. Animal studies using rats or mice as subjects.
2. Experimental studies where miRNA expression levels on the brain and/or behavior are measured.
3. Treatment and/or assessment during adolescence (PND21–PND60).
4. Assessment of stress, alcohol, and other drugs of abuse.
5. Publications in English.

The exclusion criteria were as follows:

1. Narrative reviews, systematic reviews, or meta-analyses.
2. Insufficient or deficient information on the methodology or statistics applied (number and age of subjects not reported, summary/descriptive statistics).
3. miRNA assessment is not performed on brain tissue or is not related to behavior.
4. Human studies.

### Searching Strategy and Data Extraction

The search was performed in March 2022. It covered experimental studies included in Scopus and Web of Science. The key terms applied included the following: (microRNA or miRNA or Small RNA), (adolescence), and (brain, stress, alcohol, ketamine, cocaine, nicotine, cannabis, amphetamine or PCP hydrochloride) in the title, abstract, or keywords.

Below are the specific terms used in our bibliographic search:

Scopus: TITLE-ABS-KEY (((microRNA) OR (miRNA) OR (smallRNA)) AND (adolescence) AND ((brain) OR (alcohol) OR (amphetamine) OR (nicotine) OR (cannabis) OR (ketamine) OR (PCP AND hydrochloride) OR (stress))).

Web Of Science: TS=(((microRNA) OR (miRNA) OR (SmallRNA)) AND (adolescence) AND ((brain) OR (alcohol) OR (amphetamine) OR (nicotine) OR (cannabis) OR (ketamine) OR (PCP hydrochloride) OR (stress))).

After excluding duplicates, the title and abstract were screened, and the inclusion/exclusion criteria were applied. The selected articles were read, and those meeting the criteria were used for data analysis. Data extraction was performed by three independent researchers based on a coding template that included the following items: number, species/strain, sex, and age of the experimental subject; intervention; administration procedure, doses, treatment duration, and age of intervention; miRNA and brain area evaluated, target gene and function, effect of the intervention (expression down- or upregulated), and age of the evaluation.

**Figure 1:**
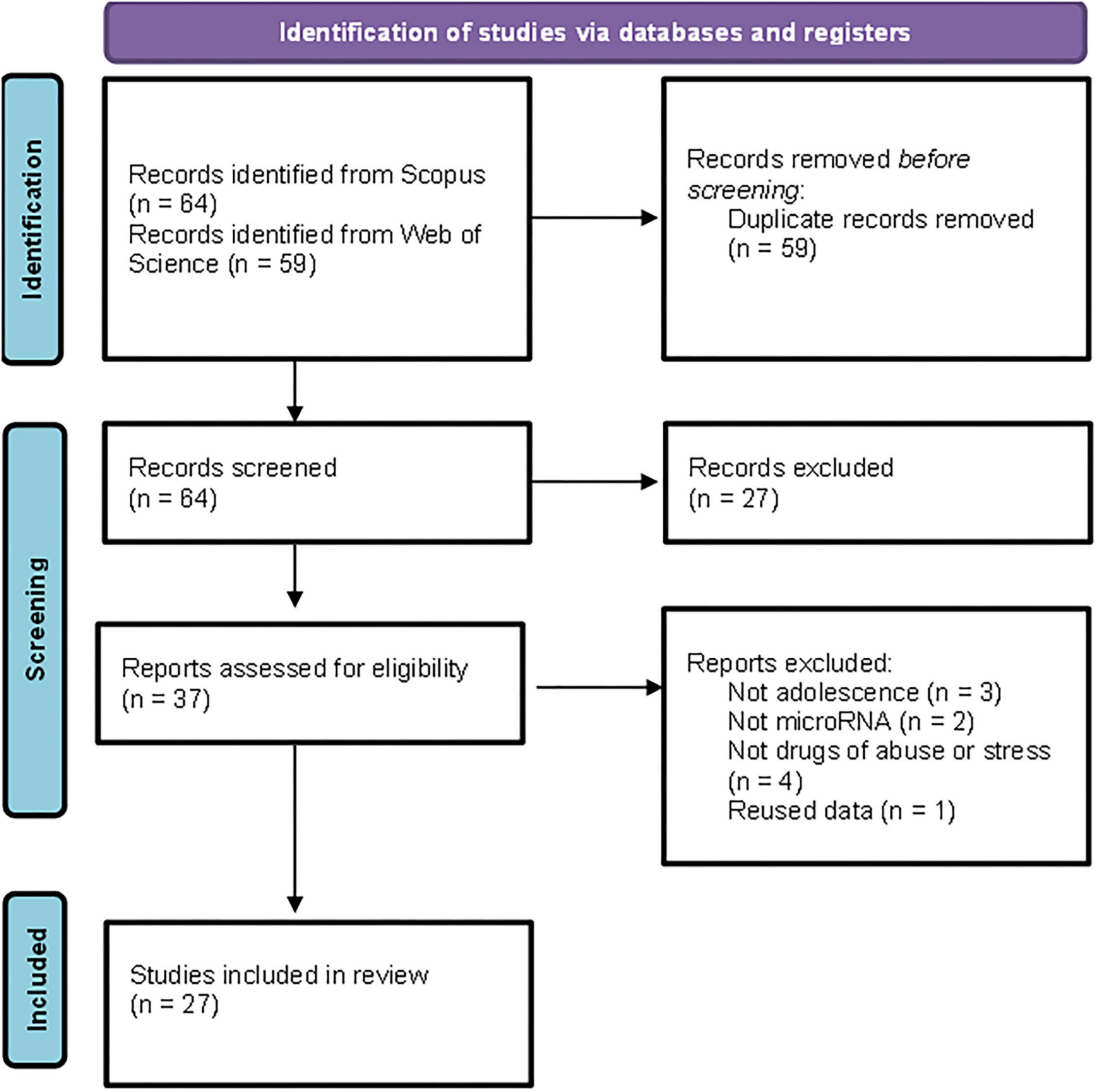
Preferred Reporting Items for Systematic Reviews and Meta-Analyses (PRISMA) flowchart illustrating the search strategy applied to select the publications included in the review.

The detailed PRISMA flowchart showing the selection procedure is presented in Figure 1. We obtained a total of 123 articles from the databases. After the exclusion of duplicates, the initial database search yielded 64 potentially relevant citations, of which 37 were retained for full-text review. Finally, 27 articles matched the inclusion criteria.

## Results

### General Description of Data

**Figure 2:**
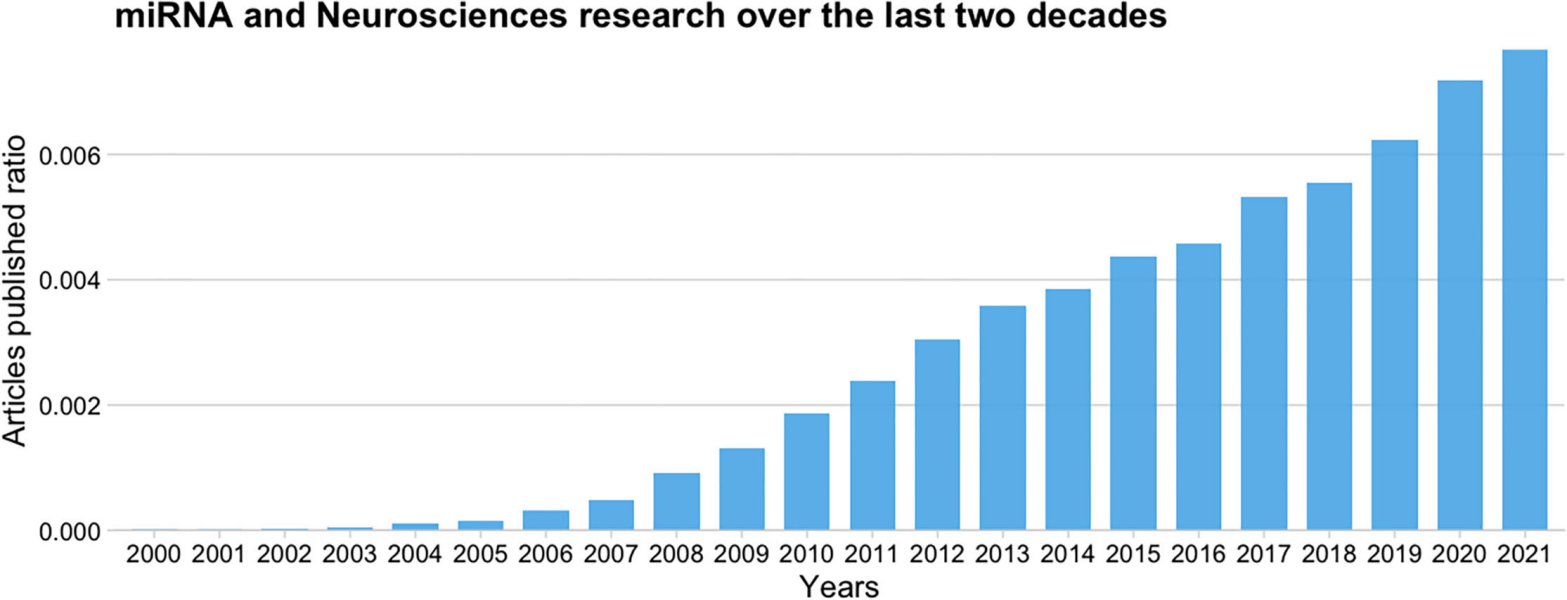
Ratio of published articles on miRNAs and neurosciences to the total number of articles published over the last 20 years according to the Europe PMC database.

Since its discovery (Lee et al., 1993), the interest in miRNAs among neuroscientists has rapidly increased. Figure 2 shows a ratio comparing the number of articles published per year over the last two decades that relate miRNAs and neurosciences [(“miRNA or miR”) and (Neuroscienc^*^)] to the total number of articles published, according to the Europe PubMed Central RESTful Web Service. We can observe that the publication rate has increased three-fold from 2010 to 2020, and it continues to grow.

Regarding the bibliographic characteristics of the 27 articles that compose this systematic review, they have been published in 23 different scientific journals. Among them, it can be highlighted as Plos One with four papers published, followed by Translational Psychiatry with two papers. Moreover, Plos ONE'S papers received a total of 107 citations, followed by Translational Psychiatry (47 citations in two articles) and Journal of Psychiatric Research (46 citations in only one article published).

**Figure 3:**
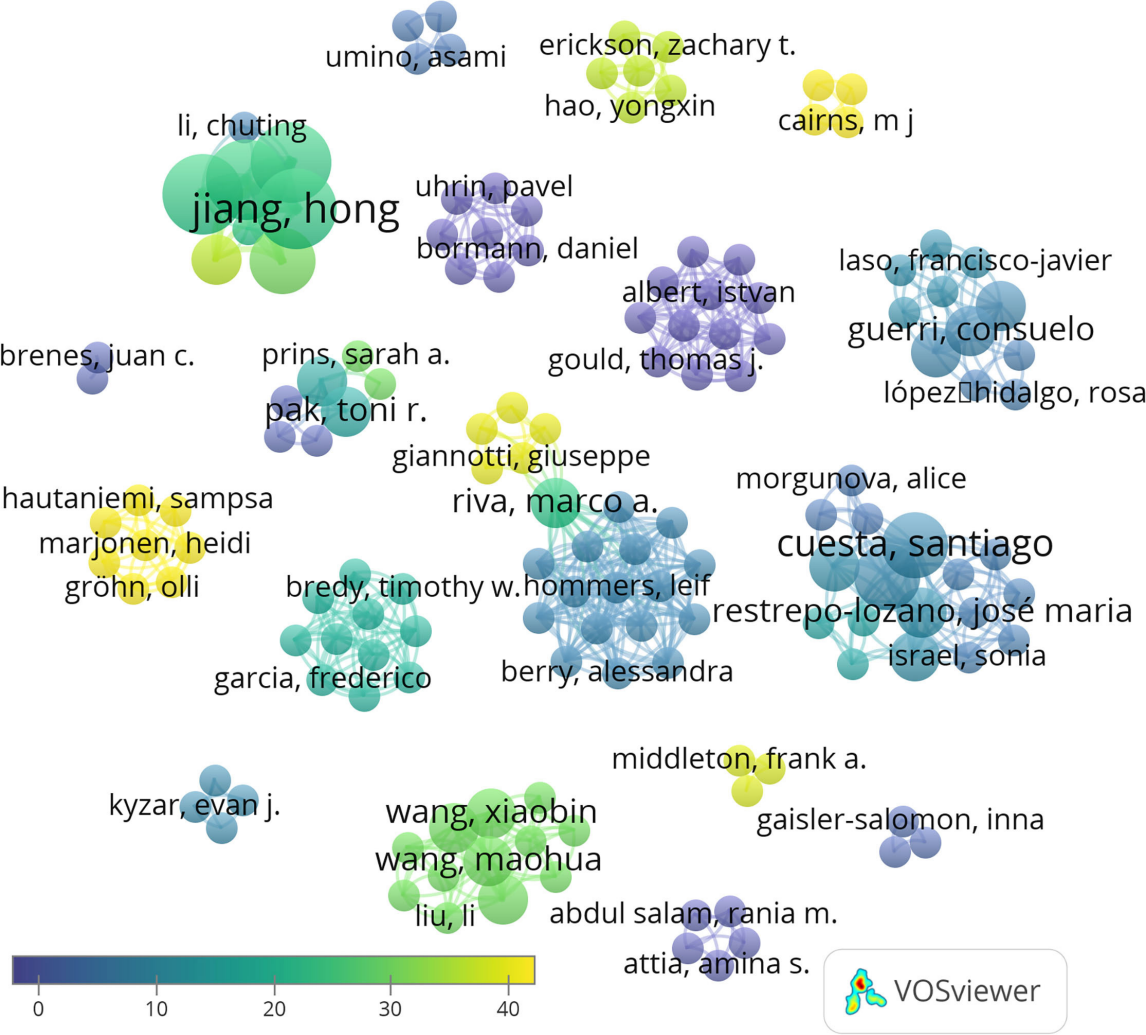
Network data map of the articles' authorship indicating research groups and collaborations. Collaboration between only two groups its shown in the center of the figure. Some representative authors of each research team are highlighted. Point size represents the number of published articles. Colors show the average citations, and lines show clusters based on co-authorship.

A total of 147 authors have been involved in the publication of these 27 articles. Hong Jiang, Dexiang Liu, Yuan Liu, and Fang Pan are the authors with more articles published on the topic, being coauthors of four papers. The analysis of the number of publications per author and their co-authorship shows 19 independent clusters. A link appears only in two of these groups of authors showed, thus indicating few collaborations between independent groups (Figure 3). This can be attributed to the fact that miRNAs are an emergent area of research in neurosciences, and the opportunities for interaction are still scarce.

**Figure 4:**
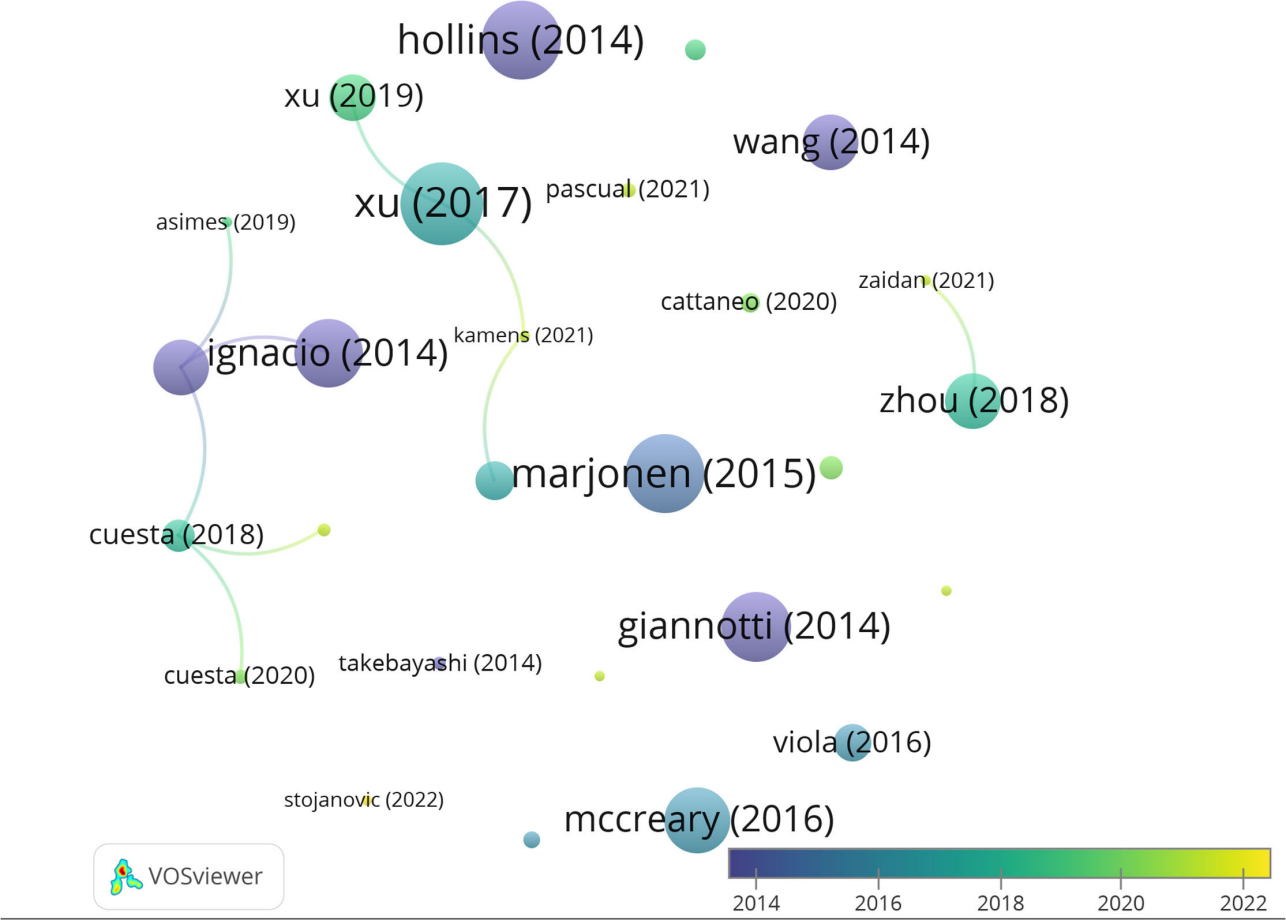
Network data map of the articles' citations. Point size represents the number of citations received and colors the publication year. Lines represent the number of co-citations evidencing only three clusters.

Taking into account the total number of citations, the most cited authors are Hong Jiang, Dexiang Liu, Yuan Liu, and Fang Pan (95 citations, average of 23.75 per paper), Jingjing Xu (88 citations, average of 29.3 per paper), and Rui Wang (69 citations, average of 34.5). Moreover, of the 27 articles included in our database, the three most cited articles are Xu et al. (2017) (46 citations), followed by Hollins et al. (2014) (44 citations), and Marjonen et al. (2015) (44 citations) (Figure 4). A total of 546 articles have been cited in the 27 papers selected, being that of Spear (2000) the most cited.

### Animals: Strain, Sex and Age

Most of the studies (18 of the 27) used rats, mainly Wistar (n = 8) and Sprague-Dawley (n = 5). Mice models were used in nine studies with C57BL/6J being the most widely used strain (n = 8). Only Stojanovic et al. (2022) used a transgenic model, miR-132/212 knockout (KO) mice.

Regarding sex, 22 of the 27 studies used only male mice, while one study focused on intergenerational effects and evaluated F0 female mice (Zaidan et al., 2021). The rest of the studies (n=4) included both male and female mice (Ignacio et al., 2014; Cattaneo et al., 2020; Ibáñez et al., 2020; Pascual et al., 2021).

With respect to the age range considered adolescence in rodents, a broad definition was applied. In addition to the strict criteria, covering from postnatal day 28 to 42 (PND-28–42), which has been considered prototypic adolescence according to biological and behavioral discontinuities (Varlinskaya and Spear, 2006), earlier ages from PND21, late adolescence extended to PND-60–70 and young adulthood have been included. Only four of the studies performed a prenatal intervention and evaluated miRNA levels during adolescence (Ignacio et al., 2014; Marjonen et al., 2015; McCreary et al., 2016; Cattaneo et al., 2020). The majority of the studies (n=22) included animals ranging from 21 to 50 days of age, and only one study (Wang et al., 2014) used PND-50–57. Regarding age at assessment, five of these studies measured miRNAs at 55 and 62 days of age (Hollins et al., 2014; Wang et al., 2014; Cattaneo et al., 2020; Ibáñez et al., 2020; Zaidan et al., 2021) and three between 70 and 102 days of age (Giannotti et al., 2014; McCreary et al., 2016; Kyzar et al., 2019). The rest of the studies also evaluated animals aged from PND21 to PND50. In addition, groups including adults, ranging from PN-90 to PND-103, were added for comparison in several studies.

### Normal miRNA Expression Profile Changes During Adolescence

Determining the miRNA levels during adolescence is essential to understanding the normal changes that take place at this stage of life and the environmental impact. Only a small number of the studies identified, however, report the evolution of miRNA expression profiles during adolescence in control non-treated animals. These studies compare miRNA expression during adolescence and adulthood. Prins et al. (2014) quantified the normal developmental expression profile of miR-10a-5p, miR-26a, miR-103, and miR-495 in the dorsal and ventral hippocampus (vHC) of male rats at three-time points (PND 30, PND 44, and PND 73). The authors found a differential expression pattern in each of them. In the dorsal hippocampus (dHC), miR-103 does not change during puberty while miR-10a-5p expression decreased significantly between early and middle puberty, and miR-26a significantly decreased only at late puberty. Also, miR-495 expression increases between early and mid/peri-puberty. Interestingly, the same miRNAs showed a different expression pattern in the vHC. Specifically, miR-10a-5p showed a significant increase by late puberty and miR-26a significantly decreased at mid/peri-puberty but this change did not persist. The levels of miR-103 and miR-495 had a similar profile consisting of a decrease during middle puberty but a recovery at late puberty. The authors reported altered hippocampal expression of the putative target genes coding BDNF (brain-derived neurotrophic factor) and SIRT1 (histone deacetylase sirtuin1), both sensitive to ethanol exposure. Likewise, the expression of miR-19a/b-3p, miR-34a, and miR-488-3p from PN30 to PN74 in the vHC was explored by Asimes et al. (2019). They found a progressive decline in miRNA expression during adolescence in control untreated rats, suggesting that their target gene expression would be increased. This seems to be related to the regulation of neurodevelopmental processes such as neurogenesis, cell migration, and synaptic plasticity. It can be proposed that the dynamic expression patterns of these genes during adolescence reflect changes in neural plasticity, learning, and memory abilities.

Cecilia Flores' group, from McGill University, also explored the normal miRNA expression profile during adolescence in two studies (Cuesta et al., 2020; Torres-Berrío et al., 2021). They consistently found that miR-218 increases significantly both blood and brain expression levels in the ventral tegmental area (VTA) and medial prefrontal cortex (mPFC) from PND21 to PND75. The authors identified as the target gene the Netrin-1 receptor, deleted in colorectal cancer (DCC) which is involved in axon guidance. DCC receptors within mesolimbic neurons play a crucial role in determining the developmental trajectories of dopamine neurons targeting mPFC, thus influencing the maturation of the reward brain system organization and its function. In fact, miR-218 has been associated with the stress response and its dysregulation has been linked with susceptibility to stress and depression.

In all, only four of the 27 studies reviewed have explored normal miRNAs' expression levels longitudinally in the course of adolescence. Most of the papers have measured these levels only at a single time point, thus avoiding comparisons with another age point. Despite this, we have identified eight papers quantifying miRNAs at different points of adolescence and/or adulthood in untreated control groups to perform comparisons with experimental treated groups. Thus, the quantifications reported are relative values that do not allow us to evaluate the course of miRNA expression profiles changes during adolescence in the absence of the treatment applied (Takebayashi et al., 2014; Li et al., 2016; Liu et al., 2016; Xu et al., 2017, 2019; Cattaneo et al., 2020; Zaidan et al., 2021; Stojanovic et al., 2022).

In conclusion, the results, although scarce, point to normal dynamic miRNA expression profiles in the developing HC and the mesolimbic reward system during adolescence which participate in the maturation of behavioral functions such as learning, memory, and emotional reactivity. These changes might contribute to the peculiar behavioral combination of novelty seeking and enhanced stress reactivity described in adolescents.

### Effect of Ethanol Exposure on miRNA Expression and Adolescence

All the studies reviewed used similar ethanol doses, ranging between 2 and 3 g/Kg, that were i.p. injected in most of the cases, although oral gavage was also used (Prins et al., 2014; Asimes et al., 2019). Only Marjonen et al. (2015) forced ethanol drinking as only 10% (v/v) ethanol solution was available. Most of the studies (5 of 7) applied ethanol intermittent exposure during adolescence with 7–8 injections on alternate days. All studies applied a strict age criterion regarding ethanol exposure and/or assessment of miRNA expression as they ranged from PND28 to PND44. Some of them identified long-lasting effects at PND58 (Ibáñez et al., 2020) and PND73-74 (Prins et al., 2014; Asimes et al., 2019), ages that can be considered late adolescence or young adulthood. Only two studies investigated the effect of prenatal alcohol exposure either drunk by pregnant mice during the first 8 gestational days (Ignacio et al., 2014) or i.p. injected into pregnant rats in GD12 (Marjonen et al., 2015).

Table 1 summarizes the reported effects of ethanol exposure on miRNA expression during adolescence. The large-scale and genome-wide (Ignacio et al., 2014; Marjonen et al., 2015) analyses of prenatal ethanol-induced changes in miRNAs confirmed and extended previous reports on models of fetal alcohol spectrum disorders (FASDs). They found several miRNAs and mRNAs changes in AM (Ignacio et al., 2014) and HC (Marjonen et al., 2015), which are related to pathways involved in the embryonic regulation of the cell cycle and cell death pathways.

**Table 1:** Effect of ethanol exposure on brain miRNA expression and adolescence.

**References**	**MicroRNA**	**Animals**	**Age**	**Brain area**	**Function**
Prins et al. (2014)	• ↓ miR-26a •↓ miR-495 •↑ miR-10a-5p •↑↓ miR-103* •↑↓ miR-495*	Wistar rats	PND 37–44	• Dorsal hippocampus •Ventral hippocampus	• Synaptic plasticity (BDNF). •Neurotransmitters and cell signaling (SIRT1). •miR biogenesis (Dicer and Drosha enzymes) •Anxiety
Ignacio et al. (2014)	• ↓ miR-1843ª-3p •↓ miR-221-5p •↓ miR-29c-3p •↓ miR-384-5p •↓ miR-412-3p •↓ miR-129-1 •↓ miR-138-2 •↓ miR-155 •↓ miR-322-2 •↑ miR-34c •↓ miR-496 •↓ miR-9a-2 •↑ miR-218a-2 •↑ let-7c-1 •↑ let-7c-2-3p •↑ miR-542-1 •↑ miR-200b-3p •↑ miR-26b-3p •↓ miR-542-2	Long-Evans rats	Prenatal	• Amygdala •Ventral striatum	Neurotransmitters and cell signaling (P53, GABAR, glutamate receptor, and CREB). •Social behavior.
Marjonen et al. (2015)	• ↑ miR-138-2 •↑ miR-290 •↓ miR-16-2	C57BL/6J Mice	Prenatal	• Hippocampus	• Synaptic plasticity. •Neurodevelopment
Asimes et al. (2019)	• ↓ miR-19a-3p •↓ miR-19b-3p •↓ miR-29a-3p •↓ miR-29c-3p •↓ miR-34a •↓ miR-488-3p	Wistar rats	PND 37–44	• Ventral hippocampus	• Neurodevelopment. Synaptic plasticity. Pubertal maturation. (ATXN1, KCNC3, VAMP2, VDAC1)
Kyzar et al. (2019)	• ↑ miR-137	Sprague-Dawley rats	PND 28–41	• Amygdala	• Neurodevelopment. Epigenetic regulation (LSD1 and Bdnf IV promoter). •Anxiety
Ibáñez et al. (2020)	• ↑↓ miR-146a-5p# •↓ miR-21-5p	C57BL/6J mice	PND 30–43	• Cerebral cortex	• Neuroinflammatory response (TLR4 Traf6, Stat3, Camk2a)
Pascual et al. (2020)	• ↑ miR-155-5p •↑ miR-96-5p# •↑ miR-182-5p#	C57BL/6J mice	PND 30–43	• Hippocampus	• Neuroinflammatory response. •Synaptic plasticity (mTOR, CREB) Learning and memory

↑ Increased in comparison with the control group; ↓ decreased in comparison with the control group; ^*^age-dependent effects; # sex-dependent effect.

Also, intermittent ethanol exposure modeling binge drinking during adolescence in male rats has been reported to alter the normal expression of several miRNAs in the dHC and the vHC. Differential effects age- and brain region-dependent were found in miR-10a-5p, miR-26a, miR-103, and miR-495 (Prins et al., 2014). Using a similar ethanol administration procedure, the same team has reported vHC altered expression only in 6 miRNAs (miR-19a-3p, miR-19b-3p, miR-29a-3p, miR-29c-3p, miR-34a, and miR-488-3p) out of a total 88 measured (Asimes et al., 2019). The effects were long-lasting as they remained at PND73-74. The reported hippocampal changes are related to sexual maturation and neurodevelopmental processes, such as neurogenesis and migration. Likewise, adolescent ethanol exposure increased miR-137 expression in the central amygdala (Kyzar et al., 2019). This is related to epigenetic alterations due to decreased expression of its target lysine-specific demethylase (LSD1) which is involved in chromatin changes at the Bdnf IV promoter, thus related to synaptic plasticity and memory. Finally, a series of reports by the same research group have demonstrated a relationship between adolescent alcohol exposure and regulation of inflammation in mice. Regarding neuroinflammation, ethanol exposure lowers cortical miR-146a-5p and miR-21-5p in female mice, thus inducing a higher expression of inflammatory target genes (Ibáñez et al., 2020). With respect to HC, this group has shown that binge-like ethanol exposure upregulates miR-96-5p and miR-182-5p in male mice but miR-155-5p in both male and female mice (Pascual et al., 2021). This is associated with dysfunction of mTOR-regulated autophagy which is involved in synaptic pruning.

In all, the results indicate a detrimental impact of ethanol on the HC and AM function mediated by dysregulation of a variety of miRNA expressions that disrupt neurodevelopmental processes such as proliferation, migration, dendritic remodeling, and synaptic plasticity and induce changes in neurotransmission. This is in accordance with the well-known learning and memory impairments as well as enhanced stress vulnerability and risk of depression associated with adolescent ethanol exposure (Crews et al., 2019).

### Drug Abuse on miRNA Expression and Adolescence

According to the inclusion criteria, we identified nine studies aimed to assess the effect of drugs of abuse other than ethanol (Table 2). They included amphetamine, cannabis, cocaine, nicotine, and phencyclidine. Amphetamine is applied in three studies (Cuesta et al., 2018; Cattaneo et al., 2020; Sequeira-Cordero and Brenes, 2021) and cocaine (Giannotti et al., 2014; Viola et al., 2016) in two of them, while we found only one paper using each of the other drugs. Although all the studies administered the drug during adolescence, some of them used extreme ages, ranging from very early adolescence such as PND21-31 (Cuesta et al., 2018; Cattaneo et al., 2020) to late adolescence at PND-50–57 (Wang et al., 2014). The majority, however, used standard age criteria, and some of them included several ages for comparison (Takebayashi et al., 2014; Stojanovic et al., 2022).

**Table 2:** Effect of drug abuse on brain miRNA expression and adolescence.

**References**	**MicroRNA**	**Drug**	**Animals**	**Age**	**Brain area**	**Function**
Giannotti et al. (2014)	• ↓ let7-d •↓ miR-124 •↓ miR-132	Cocaine	Sprague-Dawley rats	PND 28–42	Medial Prefrontal Cortex	• Synaptic plasticity
Takebayashi et al. (2014)	• ↑ miR-212* •↑ miR-132*	Phencyclidine (PCP)	Wistar rats	PND 8–50	Thalamus and Neocortex	• Neurodevelopment NMDAr (PAIP2A, H2AFZ)
Wang et al. (2014)	• ↓ miR-214	Ketamine	Sprague-Dawley rats	PND 50–57	Hippocampus	• Neuronal apoptosis (PTEN). •Learning and memory.
Hollins et al. (2014)	• ↑ miR-23a	Cannabis	Wistar rats	PND 35	Left Entorhinal Cortex	• Psychiatric diseases (schizophrenia).
Viola et al. (2016)	• ↓ miR-212	Cocaine	BALB/c mice	PND 35–44	Prefronal Cortex	• Development and neural plasticity.
Cuesta et al. (2020)	• ↑ miR-218	Amphetamine	C57BL/6J mice	PND 22–31	Ventral Tegmental Área	• Development and neural plasticity (DCC).
Cattaneo et al. (2020)	• ↑ miR-218*	Amphetamine	C57BL/6J mice	PND 22–31	Ventral Tegmental Área	• Development and neural plasticity (ROBO).
Sequeira-Cordero and Brenes (2021)	• ↑ pri-miRNA-132	Amphetamine	Wistar rats	PND 35–46	Dorsal striatum	• Neural plasticity
Stojanovic et al. (2022)	• miR-132/21	Nicotine	C57BL/6J mice; miRNA- 132/212 KO	PND 35–63	Hippocampus	• Synaptic plasticity. •Neurotransmitters and cell signaling (Ach). •Memory functions.

↑ Increased in comparison with the control group; ↓ decreased in comparison with the control group; ^*^no expression change assessed or found.

Regarding the doses, they were the same in the two studies using cocaine, but several amphetamine doses were compared, and dose-dependent effects were reported. The effective doses vary from 2.5 mg/Kg, used by Sequeira-Cordero and Brenes (2021) in rats, to 0,5 and 4 mg/Kg, applied by Cuesta and colleagues (2018, 2020) in mice. The results are consistent with the fact that amphetamine is a monoaminergic psychostimulant drug, affecting the mesocorticolimbic and nigrostriatal dopaminergic systems. On the one hand, Cuesta and colleagues found that amphetamine disrupts the normal age-dependent downregulation of miR-218 during adolescence in dopamine neurons. Thus, amphetamine (4 mg/Kg) during adolescence but not in adulthood increases miR-218 expression in the ventral tegmental area (VTA) neurons coexpressing the Netrin-1 receptor DCC. The authors demonstrate that DCC is the relevant target of miR-218 since amphetamine did not alter the expression of other receptor guidance cues such as ROBO1 (Cuesta et al., 2020). Moreover, in a later study, the same authors confirm their previous findings regarding nucleus accumbens and prefrontal cortex changes induced by high (4 mg/Kg) amphetamine dose in DCC and Netrin-1 expression. They report, however, no effect of low (0,5 mg/Kg) amphetamine doses on miR-218 expression, even though it improves performance in a go/no-go behavioral task that is impaired by high amphetamine doses. On the other hand, Sequeira-Cordero and Brenes (2021) applied amphetamine administration that increased locomotor activity in the open-field test. They reported upregulation of pri-miR-132 (primary miR-132) expression that correlated with the dopaminergic metabolite DOPAC (3,4-dihydroxyphenylacetic acid). The effect was significant in the dorsal striatum being higher than in the nucleus accumbens. The expression of miR-132 is CREB-dependent, and it is associated with the regulation of processes required for neural plasticity involving dendritic spines remodeling. Hence, although scarce, taken together, these results point to the impact of monoaminergic drugs in the expression of miRNAs both in the mesolimbic and nigrostriatal dopaminergic systems. This is consistent with the effect of cocaine administration on miRNA expression in the prefrontal cortex during adolescence. It downregulates the expression of let7d, miR-124, miR-132 (Giannotti et al., 2014), and miR-212 (Viola et al., 2016), which are involved in the regulation of Bdnf expression and neural plasticity. In the latter study, the effect on miR-212 was induced by conditioned place preference (CPP) and it was reversed by early maternal separation.

Among the reported miRNA expression's changes induced by amphetamine and cocaine, miR-132 and miR-212 deserve special mention as they seem to play a widespread role in the effect of different drugs of abuse during adolescence. In addition to the above-described changes induced by amphetamine and cocaine, the NMDA receptor antagonist phencyclidine increases the expression in the thalamus and cortex of the phencyclidine-responsive transcript *prt6*, a ncRNA that includes miR-132 and miR-212. The effect is evident during adolescence (PND32 and PND50) but not in younger rats (Takebayashi et al., 2014). The authors reported also similar effects induced by methamphetamine at PND-50. Furthermore, nicotine abolishes hippocampal LTP (long-term potentiation) in KO mice with the deletion of miR-132 and miR-212 (Stojanovic et al., 2022). Finally, other miRNAs associated with drug exposure during adolescence in rats are miR-214, which is downregulated in the hippocampus after adolescent ketamine injections that also induce learning and memory deficits (Wang et al., 2014), and miR-23a, which is upregulated in the entorhinal cortex by injections of the synthetic cannabinoid HU210 (Hollins et al., 2014).

In spite of the variety of miRNAs associated with adolescent abuse of substances, altogether the results indicate altered expression of miRNAs targeting genes involved in the development of the neurons in the mesocorticolimbic and nigrostriatal dopaminergic systems as well as in hippocampal synaptic plasticity. It can be proposed that these changes are linked to adverse effects on learning, memory, emotional regulation, and addictive behavior.

### Stress Impact on miRNA Expression and Adolescence

We identified 11 studies that explored the effect of stress on miRNA expression during adolescence. Chronic unpredictable stress (CUS), applied in five of them, was the most common procedure used to induce stress. CUS protocols included different stressors repeatedly administered in random order for 1 week (Zaidan et al., 2021), 3 weeks (Xu et al., 2017, 2019; Zhou et al., 2018), or 5 weeks (Muhammad et al., 2021; Zaidan et al., 2021). Although CUS protocols might be variable, they typically include stressors such as food and/or water deprivation, noise, pinching tail, high temperature, swimming in cold water, and sleep deprivation/circadian rhythms disturbances, and in some cases, foot shocks or social stress by crowding are added. All the CUS studies started the protocol in early adolescence (PND-28–33), thus covering the entire adolescent period, except Zaidan et al. (2021) who began at PND-45. Apart from CUS, other stress-inducing protocols applied foot shocks, restraints, and social stress. The protocol proposed as a model of posttraumatic stress disorder (PTSD) included repeated inescapable foot shocks applied at early adolescence (PND-28) for 6 consecutive days (Li et al., 2016; Liu et al., 2016). Protocols for inducing social stress varied from isolation (McCreary et al., 2016) to physical aggression by unfamiliar male mice in the chronic social defeat stress (CSDS) (Torres-Berrío et al., 2021) and chronic variable social stress (CVSS) induced by repeated rehousing with unfamiliar cage mates (Kamens et al., 2021). All were applied during adolescence except two of them that explored the effect of prenatal stress induced by maternal restrain (Cattaneo et al., 2020) and individual housing (McCreary et al., 2016). Although most of the studies evaluated the effect of early stress on the same subjects, there were studies that assessed the stress impact either in the offspring of the stressed mothers (Cattaneo et al., 2020) or in later generations (McCreary et al., 2016; Zaidan et al., 2021). In addition, the majority of the studies confirmed the efficiency of the stress protocols on behavioral tests such as open-field (OF), elevated plus maze (EPM), Morris water maze (MWM) sucrose preference test, and/or novel object recognition (NOR) tasks.

A summary of the results reported by the reviewed studies is shown in Table 3. As expected, assessment has been centered on the hypothalamic–pituitary–adrenal axis (HPA) and the amygdala (AM) responsible for the stress response as well as the hippocampus (HC) and prefrontal cortex (PFC), involved in its negative feedback regulation. Furthermore, the hippocampal functions involved in memory can be affected by neural plasticity induced by stress. Therefore, the functions of the identified miRNAs sensitive to stress during adolescence are associated with the regulation of corticotrophin-releasing factor receptor 1 (CRFR1) (Li et al., 2016; Zaidan et al., 2021), glucocorticoid receptors (GR) (Liu et al., 2016; McCreary et al., 2016; Xu et al., 2017, 2019), serotonin receptor 5-HT1AR (Liu et al., 2016) as well as those involved in developmental processes such as NT-3 (McCreary et al., 2016), the netrin-1 guidance cue receptor DCC (Torres-Berrío et al., 2021), and pathways relevant for neural plasticity.

**Table 3:** Effect of stress on brain miRNA expression and adolescence.

**References**	**MicroRNA**	**Procedure**	**Animals**	**Age**	**Brain area**	**Function**
Li et al. (2016)	• ↑ miR-34c	Electric foot shock	Wistar rats	PND 28–33	Hypothalamus	• HPA regulation (CRFR1 mRNA). •Stress response.
McCreary et al. (2016)	• ↑ miR-182 •↑ miR-10a-5p •↑ miR-124-3p	Prenatal Social isolation	Long-evans rats	Prenatal	Prefrontal cortex	• Neural plasticity (BDNF, NT-3/miR-182)
Liu et al. (2016)	• ↓ miR-135a •↑ miR-16	Electric foot shock	Wistar rats	PND 28–33	Prefrontal cortex; Hippocampus	• Neurotransmitters and cell signaling (SERT, 5HT1AR). •Depressive disorder.
Xu et al. (2017)	• ↑ miR-124a •↑ miR-18a	CUMS	Wistar rats	PND 28–49	Basolateral amygdala	• HPA regulation (GR, FKBP5). •Depressive disorder.
Zhou et al. (2018)	• ↑ miR-543-5p •↑ miR-382-3p •↑ miR-183-5p •↑ miR-3753-5p •↑ miR-410-5p •↑ miR-202-3p •↑ miR-493-3p •↓ miR-370-3p	CUMS	Sprague-Dawley rats	PND 30–45	Hippocampus	• Neurotransmitters and cell signaling (Ach, glutamate). •Depressive disorders.
Xu et al. (2019)	• ↑ miR-18a •↑ miR-124a •↓ miR-511*	CUMS	Wistar rats	PND 28–49	Prefrontal cortex and hippocampus	• Vulnerability to depressive disorder.
Cattaneo et al. (2020)	• ↑ miR-30a-5p	Prenatal restrain	Rats	Prenatal	Prefrontal CORTEX	• Psychiatric diseases.
Zaidan et al., 2021	• ↓ miR-34c	Pre-reproductive stress	Sprague-Dawley rats	Pre-reproductive	Amygdala	• Stress response.
Torres-Berrío et al., 2021	• ↑ miR-218	CSDS	C57BL/6J Mice	PND 21–75	Medial prefrontal cortex	• Neurodevelopment (DCC).
Muhammad et al. (2021)	• ↓ miR-125a-5p •↑ miR-501-3p	CUS	Wistar rats	PND 33–60	Hippocampus; cortical tissue	• Neuroinflammatory response (ET-1, ZO-1)
Kamens et al. (2021)	• ↓ miR-429-3p •↓ miR-200a-3p •↓ miR-96-5p •↓ miR-141-3p •↓ miR-200b-3p •↓ miR-183-5p •↓ miR-200a5p •↓ miR-182-5p •↓ miR-200c-3p •↓ miR-141-5p •↓ miR-183-3p •↓ miR-497b	CVSS	C57BL/6J mice	PND 25–59	Prefrontal cortex	• Neurodevelopment, Synaptic plasticity (MAPK, AMPK signaling and gap junction).

↑ Increased in comparison with the control group; ↓ decreased in comparison with the control group; ^*^ only in adults rats.

Among the variety of miRNAs reported to be associated with stress, only the expression of 3 miRNAs has been found to be altered by different stress protocols or in different brain areas. Upregulation of miR-124-a is induced by the same CUS protocol in basolateral AM (Xu et al., 2017), PFC, and HC (Xu et al., 2019). The effect is permanent lasting to adulthood, and it is associated with decreased GR levels and increased behavioral indices of anxiety. Also, independent research teams have reported that adolescent CUS induces similar upregulation of miR-183-5p in HC (Zhou et al., 2018) and PFC (Kamens et al., 2021). Finally, it is noteworthy that miR-34 is the only one described to be affected by different stress-inducing protocols. Increased expression of miR-34 has been described in response to both adolescent foot shock (Li et al., 2016) and CUS (Zaidan et al., 2021). Li et al. (2016) centered the study on the expression of miR-34c and its target CRFR1 mRNA in the rat hypothalamus. They reported that acute stress during early adolescence increased miR-34c and decreased CRFR1. Interestingly, the level of miR-34c expression returned to normal in adulthood although permanent dysregulation of the HPA with decreased CRFR1 and anxiety-like behavior in EPM and MWM persisted in adulthood. Zaidan et al. (2021) have explored the transgenerational effects of the prereproductive CUS on the miR-34 family of miRNAs. CUS applied during late adolescence induced four days later, at PND56, a selective short-term decrease of miR-34-c, but not miR-34a or miR-382 expression in the AM, while mi-34a and miR-34c decreased in oocytes and blood. In the next generations, F1 and F2, the maternal prereproductive CUS induced a miR-34a expression profile opposite in AM and PFC during adolescence but not at older ages. While miR-34a expression increased in PFC, it decreased in AM. Interestingly, the parallel changes in CRHR1 persisted into adulthood. The authors interpret this inverse pattern of changes as evidence of different developmental patterns of AM and PFC. Regarding both CRHR1 and novelty-induced behavior in OF and NOR, the effect of maternal exposure to CUS was sex-dependent. While it decreased CRHR1 and anxiogenic behavior in males, both increased in females.

Taken together, the results evidence the impact of severe stress exposure on HPA and AM that mediate the stress response as well as HC and PFC, involved in its negative feedback regulation. It is remarkable that, unlike the results reported in other sections, there is overlap between the results obtained in separate studies using different stress-inducing procedures and brain areas. This is the case for miR-129-a, miR-34, and miR-183-5p, which modulate the expression of genes such as GR or CRHR1. These findings support the fact that miRNA dysregulation during adolescence would contribute to the enhanced stress vulnerability and risk of psychiatric disorders such as schizophrenia (Thomas and Zakharenko, 2021) and depression (Morgunova and Flores, 2021; Torres-Berrío et al., 2021).

## Discussion

A general overview of the results reported by the rodent studies reviewed supports a relevant and complex role of miRNAs in the regulation of the molecular processes involved in adolescent brain and behavior development. It is noteworthy that the majority of the studies agree on applying strict age criteria to define adolescence, covering from PND-28 to PND-42 (Varlinskaya and Spear, 2006). This favors comparisons and prompts precise conclusions. Although scarce, reports including several age time points suggest a dynamic miRNAs network sensitive to environmental events with distinctive changes across adolescence. These changes are dependent on the brain area studied which is in accordance with the fact that different brain areas exhibit different maturational courses (Spear, 2013). As expected, given the increased susceptibility of the adolescent brain to stress, exposure to different stress protocols during this period has a selective impact on anxiety-like behavior and cognition which can persist into adulthood (Liu et al., 2016; Xu et al., 2017) and even across generations (McCreary et al., 2016). Interestingly, miRNA expression changes do not always parallel behavioral changes. In fact, long-lasting effects of stress-induced by foot shocks in adolescent rats are evident in adult behavior even though acute changes in miR-34c expression do not persist (Li et al., 2016). This might reflect a transient role of miR-34c mediating the critical role of environmental cues in adolescent developmental processes, such as synaptic pruning. This could sculpt and refine the brain circuits so that brain function would be permanently modified. Alternatively, the authors cannot discard the involvement of other epigenetic factors such as DNA methylation, histone acetylation, and other types of non-coding RNAs. The reported changes in the expression profiles of several miRNAs accompanying changes in behavior and brain function support, however, miRNA regulation of mRNA translation and stability is a promising mechanism linking environmental events and molecular mechanisms involved in brain maturation.

The studies reviewed have identified several miRNAs sensitive to the treatments applied in different brain areas (Tables 1–3). Most of the studies focused on one or only a few candidate miRNAs based on previous knowledge of the metabolic and signaling brain pathways relevant to the particular treatment applied. Some studies, however, are large-scale genome-wide analyses covering a high number of miRNAs (Ignacio et al., 2014; Marjonen et al., 2015). In spite of the number of miRNA changes reported, not many particular miRNAs have been found to be affected in independent studies. As mentioned above, only three miRNAs (miR-124a, miR-183-5p, and miR-34) seem to be involved in the regulation of different stress-inducing protocols. Some of them seem to play a role also in modulating the effect of exposure to substance abuse. Hence, the miR-34 family has been related not only to stress (Li et al., 2016; Zaidan et al., 2021) but also to the effects of ethanol exposure in the ventral hippocampus (Asimes et al., 2019) and the amygdala (Ignacio et al., 2014). Also, a marginal effect of ethanol exposure on the cortical expression of miR-183-5p has been reported (Ibáñez et al., 2020). Furthermore, only miR-218 has been included in all the sections reviewed. Indeed, miR-218 expression changes have been associated with stress (Torres-Berrío et al., 2021), amphetamine (Cuesta et al., 2020), and ethanol (Ignacio et al., 2014). Therefore, as these changes have been found in mPFC, VTA, and AM, respectively, miR-218 seems to have a widespread function integrating the effects of environmental challenges during adolescent brain development. The relevance of miR-218 is further confirmed by the fact that miR-218 degradation by intracerebral infusion of antimiR-218 abolished both the effects of amphetamine (Cuesta et al., 2018) and stress (Torres-Berrío et al., 2021). Therefore, although more research is needed, the available evidence suggests that miRNAs could have a relevant role in mediating interactions between different environmental influences during this critical period of brain maturation.

Regarding the specific targets of the miRNAs affected by the treatments applied, a bias cannot be excluded as assessment in most of the studies is guided by the current knowledge on the brain areas and mechanisms responsible for the response to stress and rewarding substances. Nevertheless, the results support the contribution of the identified miRNA changes to the regulation of biological pathways involved in neural plasticity and brain developmental processes. In addition to using enrichment and path analysis techniques to evaluate potential biological consequences of miRNA expression, some studies have applied additional interventions disrupting either miRNAs by antagomirs or potential mRNA targets by small-interfering RNA (siRNA). These interventions have proven to efficiently reverse the effect of adolescent ethanol exposure on miR-137 dysregulation using antagomir and siRNA of its target epigenetic enzyme Lsd1 (lysine-specific demethylase 1) (Kyzar et al. (2019)). Likewise, antagonists of the proposed protein targeted by the identified miRNAs have been applied. This has been efficient with respect to the HPA stress response. In fact, the corticotropin-releasing factor receptor (Crhr1) antagonists NBI 27914 (Zaidan et al., 2021) and CP-154 (Li et al., 2016; Liu et al., 2016) and the glucocorticoids receptor (GR) antagonist RU486 (Xu et al., 2017) reverse some of the stress exposure effects. These interventions go beyond the correlational approach, thus helping to identify the specific molecular pathway regulated by the identified miRNAs that are affected by treatments.

Although only four of the studies reviewed included male and female, sex seems to be a relevant factor that modulates the impact of ethanol during adolescence. Ibáñez et al. (2020) found lower female than male cortical levels of anti-inflammatory miRNAs (miR-146a-5p and miR-21-5p) induced by ethanol exposure in adolescent mice. Moreover, they confirmed this finding in human and female mice plasma. Sex differences in the effect of alcohol on the expression of miR-96-5p and miR-182-5p have also been reported by the same team (Pascual et al., 2021). This is in accordance with behavioral data indicating that adolescent females are more vulnerable than males to the effects of binge alcohol drinking and it can be related to hormonal regulation of miRNAs during sexual maturation. The other two papers including both sexes did not find sex-dependent differences (Ignacio et al., 2014) or focused on those miRNAs exhibiting similar expression in both sexes and different brain areas such as miR-30a-5p associated with stress (Cattaneo et al., 2020). Hence, additional research on sex-dependent effects is critical for filling the gap.

It should be noted that we have selected those results concerning the effect of stress and substances of abuse, but a few of the studies reviewed had a wider scope including interactions with other developmental threats such as those between cannabis and maternal infections (Hollins et al., 2014, 2016) and stress with cocaine (Viola et al., 2016). The results indicate that in some cases, the combined exposure is required to induce significant changes (Hollins et al., 2016) while in others the combination enhanced the effects of each treatment on the same miRNAs (Hollins et al., 2014) or in different miRNAs (Viola et al., 2016). Advancing knowledge on the epigenetic mechanisms mediating the interaction between environmental influences along the life course can open new therapeutic opportunities for intervention. First, the evidence indicating that miRNA circulating levels can reflect brain levels (Torres-Berrío et al., 2021) points to the potential use of miRNA expression as a marker of vulnerability to the effects of adolescent environmental influences. In fact, blood miR-218 expression parallels changes induced in the mPFC by CSDS. Second, interesting research lines are those exploring the effects of therapeutic interventions such as environmental enrichment (McCreary et al., 2016), the antidepressant fluoxetine (Zaidan et al., 2021), and antipsychotic drugs including haloperidol (Takebayashi et al., 2014) or lurasidone (Cattaneo et al., 2020) to counteract or reverse the impact of treatments disrupting miRNA expression.

In all, despite the complexity of the epigenetic mechanisms involved in adolescent brain development, the reviewed studies highlight the rapidly growing utility of animal research in advancing knowledge on the emerging miRNA field. Therefore, the results reviewed represent a great contribution to understanding the particular role of miRNAs as part of the epigenetic mechanisms mediating the required impact of the particular environment on brain development during the critical adolescent period. The interest in the influence of high levels of stress and drugs or alcohol exposure on brain development is based on the reported interaction between them that might increase the deleterious long-term effects described in adult brain function and behavior. The results encourage research in this field with special interest in potential mechanisms involved in psychopathological disorders emerging during adolescence. In fact, exposure to stress and drug abuse during adolescence are recognized as risk factors for the onset of psychiatric disorders and drug addiction, thus representing serious health concerns. Animal models of depression, schizophrenia, and posttraumatic stress disorder represent a useful tool for developing preventive and therapeutic treatments.

## Data Availability Statement

The original contributions presented in the study are included in the article/supplementary material, further inquiries can be directed to the corresponding author.

## Author Contributions

All authors listed have made a substantial, direct, and intellectual contribution to the work and approved it for publication.

## Funding

This study was funded by the research projects PID2020-114269GB-I00 (MICIU, Spain), BSEJ.514.UGR20 (FEDER, Junta de Andalucía, Spain), and a predoctoral fellowship to AV-Á (FPU18/05012, MIU, Spain).

## Conflict of Interest

The authors declare that the research was conducted in the absence of any commercial or financial relationships that could be construed as a potential conflict of interest.

## Publisher's Note

All claims expressed in this article are solely those of the authors and do not necessarily represent those of their affiliated organizations, or those of the publisher, the editors and the reviewers. Any product that may be evaluated in this article, or claim that may be made by its manufacturer, is not guaranteed or endorsed by the publisher.
